# The Contribution of Proton-Donor pKa on Reactivity Profiles of [FeFe]-hydrogenases

**DOI:** 10.3389/fmicb.2022.903951

**Published:** 2022-09-28

**Authors:** Effie C. Kisgeropoulos, Vivek S. Bharadwaj, David W. Mulder, Paul W. King

**Affiliations:** National Renewable Energy Lab, Biosciences Center, Golden, CO, United States

**Keywords:** [FeFe]-hydrogenase, proton-coupled electron transfer, enzymatic reactivity, H-cluster, pKa and proton transfer, catalytic bias

## Abstract

The [FeFe]-hydrogenases are enzymes that catalyze the reversible activation of H_2_ coupled to the reduction–oxidation of electron carriers. Members of the different taxonomic groups of [FeFe]-hydrogenases display a wide range of preference, or bias, for H_2_ oxidation or H_2_ production reactions, despite sharing a common catalytic cofactor, or H-cluster. Identifying the properties that control reactivity remains an active area of investigation, and models have emerged that include diversity in the catalytic site coordination environments and compositions of electron transfer chains. The kinetics of proton-coupled electron transfer at the H-cluster might be expected to be a point of control of reactivity. To test this hypothesis, systematic changes were made to the conserved cysteine residue that functions in proton exchange with the H-cluster in the three model enzymes: CaI, CpII, and CrHydA1. CaI and CpII both employ electron transfer accessory clusters but differ in bias, whereas CrHydA1 lacks accessory clusters having only the H-cluster. Changing from cysteine to either serine (more basic) or aspartate (more acidic) modifies the sidechain pKa and thus the barrier for the proton exchange step. The reaction rates for H_2_ oxidation or H_2_ evolution were surveyed and measured for model [FeFe]-hydrogenases, and the results show that the initial proton-transfer step in [FeFe]-hydrogenase is tightly coupled to the control of reactivity; a change from cysteine to more basic serine favored H_2_ oxidation in all enzymes, whereas a change to more acidic aspartate caused a shift in preference toward H_2_ evolution. Overall, the changes in reactivity profiles were profound, spanning 10^5^ in ratio of the H_2_ oxidation-to-H_2_ evolution rates. The fact that the change in reactivity follows a common trend implies that the effect of changing the proton-transfer residue pKa may also be framed as an effect on the scaling relationship between the H-cluster di(thiolmethyl)amine (DTMA) ligand pKa and *E*_m_ values of the H-cluster. Experimental observations that support this relationship, and how it relates to catalytic function in [FeFe]-hydrogenases, are discussed.

## Introduction

**Figure 1:**
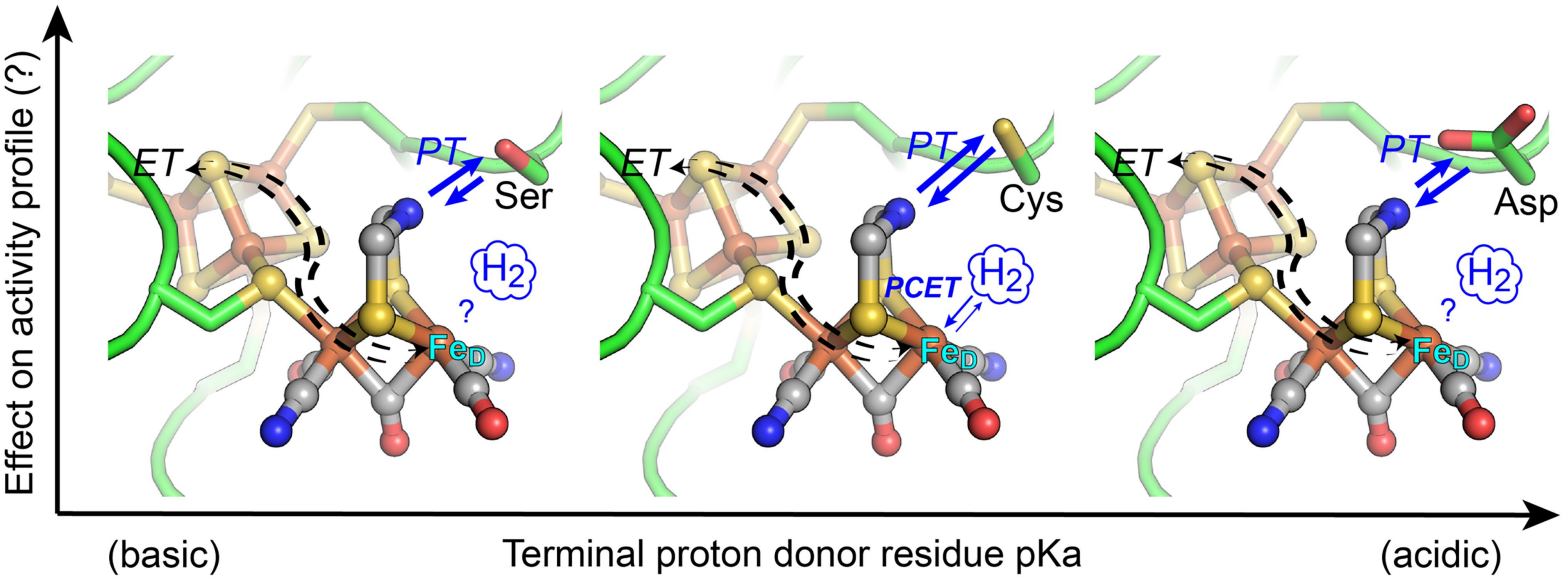
Structural depiction of the [FeFe]-hydrogenase H-cluster active-site where coordination of orthogonal electron transfer (ET) and proton transfer (PT) pathways by proton-coupled electron transfer (PCET) at the distal Fe atom (Fe_D_) of [2Fe]_H_ results in catalytic activation of H_2_. In this work, the conserved Cys residue (middle panel), which functions in proton exchange with the H-cluster di(thiolmethyl)amine (DTMA) ligand, was changed to either Ser (more basic pKa, left) or Asp (more acidic pKa, right). The activity profile of enzyme variants was measured to ascertain the extent that engineered variations to the pKa of the exchange step alter reactivity of [FeFe]-hydrogenases. Blue arrows indicate relative changes in the proton transfer direction bias for the variants. Structural representation from PDB 3C8Y, with substitution of Ser or Asp by alignment to PDB 6GLZ.

The [FeFe]-hydrogenase class of enzymes fulfill significant roles in H_2_ metabolism and energy transduction. They catalyze the reversible reaction, H_2_↔2H^+^ + 2e- by performing either H_2_ gas evolution (i.e., proton reduction) or H_2_ oxidation. This is mediated by a complex metallocofactor, or H-cluster, consisting of a diiron site ([2Fe]_H_) coordinated by a conserved cysteine to a [4Fe-4S] cubane ([4Fe-4S]_H_; Figure 1). To achieve this reversibility, the enzyme must couple dynamic fluxes in both PT and ET to catalytic H_2_ activation. As a result, the catalytic function of the H-cluster must be adapted to a changing energy landscape. [FeFe]-hydrogenases therefore represent an ideal model system for understanding the mechanisms that enzymes employ to control proton-coupled electron transfer (PCET) at metal sites to accomplish chemical transformation reactions. While most [FeFe]-hydrogenases possess neutral reactivity profiles, i.e., similar rates for both H_2_ evolution and H_2_ oxidation, there are instances where reactivity for either one or the other is favored. For example, the overall catalytic preference across the diversity of [FeFe]-hydrogenase from *Clostridium pasteurianum* spans an impressive seven orders of magnitude from CpI (neutral reactivity) to CpII (biased toward H_2_ oxidation) and CpIII (biased toward H_2_ evolution; Adams, 1990; Poudel et al., 2016; Artz et al., 2020b). The wide range in reactivity stands in contrast to the fact that all [FeFe]-hydrogenases studied so far share a common H-cluster cofactor.

Diversity in the amino acids that comprise the catalytic site environments, differential stabilization of catalytic intermediates, amino acid composition of proton transfer pathways, and differences in thermodynamic profiles of electron transfer relays have all been proposed to account for the observed differences in reactivity profiles of [FeFe]-hydrogenases (Cornish et al., 2011; Hexter et al., 2012; Ginovska-Pangovska et al., 2014; Artz et al., 2017; Caserta et al., 2018; Duan et al., 2018; Gauquelin et al., 2018; Rodríguez-Maciá et al., 2019; Senger et al., 2019; Lampret et al., 2020). It is conceivable these properties converge to control PCET chemistry at the H-cluster, and changes in the coupling of protons and electrons at the H-cluster may also have a role in the control of reactivity. Computational and experimental studies have shown that PT flux to the H-cluster involves defined, well-conserved structural elements (Long et al., 2014; Duan et al., 2018; Lampret et al., 2020) including a strictly conserved cysteine residue that forms an H-bonding network with the di(thiolmethyl)amine ligand (DTMA) and distal Fe (Fe_D_) of [2Fe]_H_ in catalytically active enzymes (Figure 1). Cysteine is a relatively basic residue (free cysteine pKa ~8) and mediates the proton exchange step with the H-cluster during catalysis. Electron transfer can also be a control point of catalytic reactivity due to variability in midpoint-potentials of accessory iron–sulfur clusters (referred to as F-clusters) and/or their electronic interactions with the H-cluster (Adams et al., 1989; Mulder et al., 2013; Artz et al., 2017; Rodríguez-Maciá et al., 2017, 2019, 2020; Caserta et al., 2018). Together these mechanisms exert control over the PT and ET fluxes in the enzyme and contribute to the control of the PCET kinetics at the H-cluster. For example, the fine-tuning of the thermodynamic equilibria between reaction intermediates (Artz et al., 2017), H-bonding effects on the electronic structure of the H-cluster (Mulder et al., 2014; Pham et al., 2018), or matching of proton transfer pKa values to the H-cluster DTMA ligand pKa (Lampret et al., 2020; Birrell et al., 2021) are all mechanisms that have been demonstrated to influence the catalytic cycle and overall reactivity profile of the [FeFe]-hydrogenases.

To address understanding of the PCET mechanism in [FeFe]-hydrogenases, this work summarizes the results on testing the hypothesis that the pKa of the conserved residue that mediates the coupling of PT to ET (or PCET) at the H-cluster can be tuned to control reactivity in [FeFe]-hydrogenases. Using [FeFe]-hydrogenases that differ in their intrinsic reactivity, we applied reverse engineering to replace the conserved cysteine with either a more basic (serine) or acidic (aspartate) residue (Figure 1). The enzymes examined include CaI from *Clostridium acetobutylicum* (neutral reactivity) and CpII from *C. pasteurianum* (H_2_ oxidation bias), both of which have accessory F-clusters with different thermodynamic profiles (Adams et al., 1989), and HydA1 from *Chlamydomonas reinhardtii* (CrHydA1, neutral reactivity), which possesses only the catalytic H-cluster. Computational modeling was used to evaluate the pKa’s of the native and enzyme variants, and the H_2_ oxidation and evolution rates were measured to assess the reactivity profiles. The results of this work, in the context of previous biophysical studies of the reaction intermediates and redox profiles of related variants, are integrated into an overall thermodynamic model of reactivity control in [FeFe]-hydrogenase.

## Materials and Methods

### Expression, Purification, and Activity Measurements of [FeFe]-hydrogenases

CaI, CpII, and CrHydAI [FeFe]-hydrogenases were expressed and purified as previously described (King et al., 2006; Mulder et al., 2014; Ratzloff et al., 2018; Artz et al., 2020b) and assayed for both H_2_ evolution and H_2_ oxidation (uptake) using standard biochemical assays (Duan et al., 2018; Artz et al., 2020b).

H_2_ production was measured by gas chromatography (Agilent Technologies) using methyl viologen (MV) as the electron donor. The 2 ml reactions were set up in 13 ml anaerobic vials containing 1 μg to 1 mg of enzyme in 50 mM Tris (pH 8–8.3), 200–300 mM NaCl, 5% glycerol, and 10–100 mM sodium dithionite (DT); enzyme concentrations were varied as needed to measure kinetic parameters. Reactions were carried out at 37°C and initiated by the addition of MV to a final concentration of 5–80 mM. The reported rates were measured in the initial linear phase (varying from 5 to 40 min for the native enzymes and enzyme variants) of the reaction.

H_2_ oxidation was monitored by UV–Vis using either methylene blue (MB) monitored at 664 nm (ε = 95,000 M^−1^ cm^−1^) or benzyl viologen (BV) monitored at 600 nm (ε = 10,000 M^−1^ cm^−1^) as the electron acceptor (Cenens et al., 1988; Dörner and Boll, 2002; Duan et al., 2018). The 2 ml reactions were set up in 4 ml, septa-sealed cuvettes, with 1 μg to 1 mg of enzyme at various concentrations in 50 mM Tris pH 8–8.3, 300 mM NaCl, and 5% glycerol. The cuvettes were then either sparged continuously under H_2_ for 5 min, or subjected to 10 vacuum/refill cycles with H_2_ on a Schlenk line, and allowed to incubate under an overpressure of H_2_ at room temperature for 5–10 min. The reaction was then initiated with the addition of the redox dye, to a final concentration of 38 μM (MB) or 10 mM (BV). No reduction of either dye was observed when added to cuvettes containing only buffer. The *V*_max_ values for CaI, CpI, and CrHydA1 are similar when either MV or MB is used as the acceptor (Adams and Mortenson, 1984; Artz et al., 2020b). The values for CpII are maximal using MB as the acceptor (Adams and Mortenson, 1984); therefore, MB was used for all CpII H_2_ oxidation assays. Maximal rates were calculated over the initial 1–5 min of the reaction.

### Structural Models and pKa Calculations of [FeFe]-hydrogenase Variants

Since experimentally characterized and holo-structures of CrHydA1, CpII, and CaI are not yet available, the open-source neural-network-based protein prediction tool AlphaFold2 was used for their structure prediction (Jumper et al., 2021). For each protein, only the amino acid sequence was used as input to AlphaFold2 and five models were generated. Since Alphafold2 does not incorporate ligands into its structure predictions, the top-ranked AlphaFold2 predicted structure for each [FeFe]-hydrogenase was aligned with the experimental X-ray crystal structure for CpI (PDB ID: 3C8Y) for the incorporation of the H-cluster into the predicted structures. The H-cluster-bound top-ranked models for each [FeFe]-hydrogenase were then used for pKa estimations for its titratable residues using the Propka ver. 3.1 software package (Bas et al., 2008; Søndergaard et al., 2011). Of particular interest are the pKa predictions for the conserved cysteine residue (and aspartic acid for the C→D variant) that functions in proton exchange with the H-cluster. Since Propka does not consider residues with bulk pKa values >10 as titratable, values for the serine residue in the C→S variants were not predicted in this study, and experimental values from related model systems were used (Bruice et al., 1962; Liepinsh and Otting, 1996). It may be noted that these pKa calculations are intended as estimates and not exact determinations of the pKa values. This will require more computationally intensive techniques such as constant pH molecular dynamics simulations which are not undertaken in this study.

## Results

### pKa Calculations and Activity Profiles of Native [FeFe]-hydrogenases

The strictly conserved cysteine residue of catalytically active [FeFe]-hydrogenases (protein sequence number C298 CaI, C169 CpII, and C169 CrHydA1) is known to function in mediating the exchange of protons between the conserved proton transfer pathway and the H-cluster during catalysis (Cornish et al., 2011; Ginovska-Pangovska et al., 2014; Mulder et al., 2014; Lampret et al., 2020). The free-energy of proton transfer, Δ*G*^PT^, is related to the pKa of the exchange site by Equation 1;


(1) $$\Delta G^{\mathrm{PT}}=2.303\cdot \mathrm{RT}\cdot \mathrm{pKa}$$

To examine the extent that the proton-transfer residue pKa controls H-cluster protonation and enzyme reactivity (Figure 1), we first evaluated the cysteine pKa values using computational approaches and established a baseline of activity values for the model [FeFe]-hydrogenases, CaI, CpII, and CrHydA1 (Table 1). Proton-transfer residue pKa values were calculated using Propka (Olsson et al., 2011; Søndergaard et al., 2011), a computational treatment for the empirical prediction of pKa values which also takes into account the influences of the protein environment (*vide infra*). For the [FeFe]-hydrogenases listed in Table 1, the determined cysteine pKa values ranged 11.5–12. It may be noted that the calculated values are more basic compared to the pKa value for a cysteine residue in bulk solution. This can be attributed to the fact that Propka considers the contributions from desolvation energies, H-bonding energies, electrostatic reorganization energies, and coulombic interactions that are all cumulatively added to the bulk pKa value of a given titratable residue. A greater degree of H-bonding shifts the pKa to be more basic, while a greater degree of desolvation makes conjugate bases more basic and conjugate acids more acidic (Bas et al., 2008). For the conserved cysteine in [FeFe]-hydrogenases, these collective effects contribute to an overall more basic pKa value.

**Table 1:** Overview of WT [FeFe]-hydrogenase activity^a^ profiles.

Enzyme	^b^H_2_ oxidation Activity	^c^H_2_ evolution Activity	Oxidation/Evolution ratio	Calc. Cysteine pKa	^d^*E*_m_ (mV) H_ox_/H_red(H+)_	References
CpII	110,000^(MB)^	16	6,900	11.7	−410	Artz et al., 2020b
	34,000^(MB)^	10	3,400			Adams, 1990
	17,600^(MB)^	3.5	5,000			Chen and Blanchard, 1984
CpI	24,000^(MB)^	5,500	4	11.5	−400	Adams, 1990
	14,000^(MV)^	4,000	4			Adams and Mortenson, 1984
CaI	10,057^(MV)^	2,234 ± 214	5	11.6	NA	Girbal et al., 2005 This work
CrHydA1	18,375^(BV)^	1,000	18	12.0	−400 (−362)	Duan et al., 2018 Yacoby et al., 2012

a Activity reported as *μ*mol H_2_/min/mg enzyme for either H_2_ oxidation or evolution.

b The *V*_max_ values for CpII H_2_ oxidation assays are measured using 38 μM methylene blue (MB, *E*_m_ = +11 mV) as the acceptor (Adams, 1990). The *V*_max_ value of 10,057 for CaI was measured with methyl viologen (MV) *E*_m_ = −440 mV (Girbal et al., 2005), and the value of 18,357 for CrHydA1 (Duan et al., 2018) was measured with benzyl viologen (BV) *E*_m_ = −350 mV and at pH 10.

c H_2_ evolution rates obtained using reduced MV (5–10 mM) as the electron acceptor. The CaI value of 2,234 ± 214 is from this work; the CrHydA1 value of 1,000 is from (Yacoby et al., 2012). The *K*_m_ values for MV are: CpII, 0.3 mM (Adams, 1990); CpI, 6 mM (Adams and Mortenson, 1984; Adams, 1990); CaI, 0.6–1 mM (Girbal et al., 2005); and CrHydA1, 0.8–0.9 mM (Von Abendroth et al., 2008).

d *E*_m_ is defined as the H_ox_/H_red_ or H_ox_/H_red_H+ redox couples, which are not experimentally distinguished. CpI/CpII *E*_m_ values at pH 8 (Adams, 1990); CrHydAI *E*_m_ −400 mV at pH 8 (Silakov et al., 2009), or − 362 mV at pH 8 (Sommer et al., 2017).

Table 1 also shows H_2_ evolution and H_2_ oxidation rates for the model [FeFe]-hydrogenases. The corresponding reactivity preference, also referred to as catalytic bias (Abou Hamdan et al., 2012; Artz et al., 2020b; Fourmond et al., 2021; Mulder et al., 2021), can be discerned from the ratio of H_2_ oxidation activity to H_2_ evolution activity. A ratio approaching 1 is representative of the energy landscapes of the enzyme being leveled so that it no longer catalytically favors one direction or another. In such a case, the enzyme is described as having a “neutral” catalytic bias. As shown in Table 1, the dye-assays show that CaI, CpI, and CrHydA1 each have an H_2_ oxidation-to-H_2_ evolution ratio of <18, with CaI and CpI being slightly more neutral in bias than CrHydA1.

In contrast, and as shown previously (Adams and Mortenson, 1984; Chen and Blanchard, 1984; Adams, 1990; Artz et al., 2020b), CpII has a large bias toward H_2_ oxidation, with an H_2_ oxidation-to-H_2_ evolution ratio that is two orders of magnitude greater than for CaI, CpI, or CrHydA1 (Table 1). It is noted that the calculated cysteine pKa values for all the enzymes examined here are generally basic and that all the catalytic [FeFe]-hydrogenases incorporate cysteine at the exchange site (an exception to cysteine occurs in the sensory [FeFe]-hydrogenases, see Poudel et al., 2016; Chongdar et al., 2018; Fasano et al., 2021). Thus, it does not appear that the pKa of the exchange step is used to control the reactivity or catalytic bias. The fact that the relative oxidation/evolution reaction profiles differ by as much as 10^3^, with cysteines having similar pKa values, implies other factors, such as F-cluster reduction potentials, dynamic secondary interactions, and local electrostatics around the H-cluster (Adams et al., 1989; Caserta et al., 2018; Artz et al., 2020b), might be critical to the control of reactivity between enzyme types. It is also noted that the *E*_m_ of the H_ox_/H_red_ transition is similar in value for CpI, CpII, and CrHydA1 (Adams et al., 1989; Silakov et al., 2009) and near the value of the H^+^/H_2_ couple (−413 mV vs. SHE at pH 7, 1 atm H_2_), which is consistent with the minimal overpotential requirement of these enzymes for catalysis.

### Cys→Ser Variant pKa and Activity Profiles

To probe how differences in reactivity of CaI, CpII, and CrHydA1 are influenced by the pKa of the nearby proton donor residue, we examined enzyme variants where the cysteine residue is either altered to a more basic serine (C→S) or more acidic aspartate (C→D) residue. Previous site-saturation studies at the cysteine position have shown that these two mutations retain H-cluster cofactor incorporation with decreased H_2_ evolution activity (Knörzer et al., 2012; Morra et al., 2012; Mulder et al., 2014; Duan et al., 2018); however, the effects on H_2_ oxidation and full activity profiles in the variants are less understood.

The Propka method used to calculate cysteine pKa values is not configured to calculate serine pKa values (see the section Materials and Methods for a detailed discussion). However, experimental measurements of the pKa of -OH moieties in mimics of the catalytic triad in chymotrypsin assign the pKa of serine to be ~13.6 (Bruice et al., 1962). In this enzyme, the serine-OH group is deprotonated by the Nε atom of a nearby histidine during catalytic esterification of aromatic amino acids (Bruice et al., 1962; Frey, 2001). The basicity of the histidine Nε atom, with a pKa of 12, is within the pKa range of the H-cluster DTMA ligand that was determined from the modeling of FTIR spectro-electrochemical data from CrHydA1 collected at different pH values (H_red_/H_red_H^+^ pKa = 7.2 and, H_sred_/H_sred_H^+^ pKa = 11.6; Sommer et al., 2017; Birrell et al., 2021). Based on these similarities to [FeFe]-hydrogenase, we have tentatively assigned the serine residue as having a pKa of ≥13.6. Other experimental studies have measured the pKa of the hydroxyl group on a serine amino acid to be >16 using NMR (Liepinsh and Otting, 1996), further indicating its highly basic nature.

Using redox-dye mediated assays on the purified CrHydA1 C169S variant, H_2_ oxidation and evolution activities were determined and compared to CaI C298S (Cornish et al., 2011) and CpII C169S (Artz et al., 2020a; Table 2). The reactivity results show a collective shift toward H_2_ oxidation reactivity for all the enzymes. Whereas CpII C169S maintained the highest H_2_ oxidation preference among the C→S variants, the reactivity preference of CaI C298S likewise shifted over 100-fold toward H_2_ oxidation compared to WT CaI. It is also noted that while all C→S variants exhibit a decrease in the absolute reactivity rates compared to WT counterparts, this decrease is consistently more pronounced for the H_2_ evolution rates (Table 2), as has been observed for CpI (Cornish et al., 2011). This can be viewed as consistent with the basic pKa shift in relation to native Cys for the PT exchange step, and the transfer of protons from the H-cluster being more favored (discussed in more detail below).

**Table 2:** Activity^a^ profiles of [FeFe]-hydrogenase C→S variants.

Enzyme	^b^H_2_ oxidation activity	% of WT	^c^H_2_ evolution activity	% of WT	Oxidation/Evolution	Serine pKa^d^	References
CpII C169S	15,000^(MB)^	14%	0.2	1.3%	75,000	≥13.6	Artz et al., 2020a
CaI C298S	1,600^(BV)^	16%	1.2 ± 0.2	0.05%	1,300	≥13.6	Cornish et al., 2011, This work
CpI C299S	ND	ND	1.05	0.03%	–	≥13.6	Duan et al., 2018
CrHydA1 C169S	0.80 ± 0.1^(BV)^	0.004%	0.02 ± 0.01	0.002%	40	≥13.6	This work
	ND	ND	0.92	0.1%	–		Duan et al., 2018

a Activity reported as *μ*mol H_2_/min/mg enzyme for either H_2_ oxidation or evolution.

b H_2_ oxidation rates obtained at pH 8–8.3 using the redox dyes indicated in superscripts as electron acceptors: MB = methylene blue (38 *μ*M), or BV = benzyl viologen (10 mM). CaI C298S H_2_ oxidation with BV, extrapolated from value of 16% measured for CpI C299S compared to WT (Cornish et al., 2011).

c H_2_ evolution rates obtained using 10–80 mM reduced MV as the electron donor. The value for CaI C298S is from this work.

d Estimated based on Bruice et al. (1962).

### Cys→Asp Variant pKa and Activity Profiles

The effect of introducing a more acidic proton donor residue near the H-cluster in CaI, CpII, and CrHydA1 was also examined using the C→D variant (Table 3). In contrast to the C→S variant, it could be expected that the more acidic pKa of the carboxyl group (pKa ≈ 4–6, Table 3) would cause a shift in the reactivity profile toward H_2_ evolution, resulting in oxidation/evolution ratios below 1. CrHydA1 C169D displayed a ratio <1 (oxidation/evolution ratio = 0.3) although this still falls within the definition for neutral bias given above, while CaI C298D also retains a neutral bias (Morra et al., 2012). Remarkably, CpII C169D had neutral reactivity, with an oxidation/evolution ratio of 11 (Table 3).

**Table 3:** Activity^a^ profiles of [FeFe]-hydrogenase C→D variants.

Enzyme	^b^H_2_ oxidation Activity	% of WT	^c^H_2_ evolution Activity	% of WT	Oxidation/Evolution	Calc. Aspartate pKa	References
CpII C169D	19.5 ± 3.6^(MB)^	0.02%	1.8 ± 0.4	11%	11	4.1	This work
CaI C298D	433^(MV)^	4%	230	10%	2	4.3	Morra et al., 2012
CrHydA1 C169D	41 ± 17^(BV)^	0.2%	151 ± 41	15%	0.3	6.6	This work
	225^(BV)^	1.2%	582	58%	0.4		Duan et al., 2018

a Activity reported as *μ*mol H_2_/min/mg enzyme for either H_2_ oxidation or evolution.

b H_2_ oxidation rates were measured at pH 8–8.3 using the redox dyes indicated in superscripts; MB = methylene blue (38 *μ*M), MV = methyl viologen, BV = benzyl viologen (10 mM).

c H_2_ evolution rates were obtained using 5–10 mM MV as the electron donor.

Although the WT activity profiles of CaI and CrHydAI are also considered neutral, the H_2_ oxidation/evolution values for their C→D variants decreased relative to the WT, indicating a skewing toward H_2_ evolution that was not present before. This is much more striking in the case of CpII, where the H_2_ oxidation/evolution ratio for the C169D variant is shifted three orders of magnitude from WT in the direction of H_2_ evolution, effectively neutralizing the CpII bias toward H_2_ oxidation. As observed for the C→S variants, there is an overall decrease in the absolute H_2_ reactivity rates of the C→D variants compared to WT counterparts, with the decrease now consistently more pronounced for the H_2_ oxidation rates. From this, it appears that the pKa shift of the C→D substitution provides an overall leveling of the catalytic bias (oxidation/evolution ratio approaching 1).

## Discussion

Here, we have tested the prediction that reactivity in [FeFe]-hydrogenases can be tuned through perturbing the pKa value of the amino acid in the secondary coordination sphere that mediates the proton exchange step with the H-cluster. Modulation of the proton exchange site pKa was achieved by mutation of Cys and led to pronounced shifts in the reactivity profiles of all the [FeFe]-hydrogenases that were tested, regardless of the presence of F-clusters or the intrinsic bias (Table 4; Figure 1). The native [FeFe]-hydrogenases establish a common baseline for comparison between the different enzymes as they exhibit similar properties, such as the pKa of the conserved cysteine residue (pKa = 11.5–12) and the *E*_m_ for the H_ox_/H_red_ couple, which is relevant to the initial step for proton binding or H_2_ activation (CpII, −410 mV; CpI, −400 mV; and CrHydAI, −400 mV; Adams, 1990; Silakov et al., 2009). The observed trends can be summarized as follows: the C→S substitution resulted in a more basic pKa that shifted reactivity toward H_2_ oxidation, while the C→D substitution resulted in a more acidic pKa that shifted the reactivity toward that of H_2_ evolution, resulting in an overall neutralization of the catalytic bias.

**Table 4:** Summary of exchange site pKa on [FeFe]-hydrogenase activity^a^ profiles.

Enzyme	pKa^b^	Oxidation/Evolution Reactivity Ratio	^c^Fold-Change Versus WT	^d^*E*_m_ (mV) H_ox_/H_red(H+)_
CpII C169S	≥13.6	75,000	11^(Ox)^	NA
Ca1 C298S	≥13.6	1,300	260^(Ox)^	NA
CrHydA1 C169S	≥13.6	40	2.2^(Ox)^	−283
CpII	11.7	6,900	1	−410
CaI	11.6	5	1	NA
CrHydAI	12.0	18	1	−400 (−362)
CpII C169D	4.1	11	627^(Evol)^	NA
CaI C298D	4.3	2	2.5^(Evol)^	NA
CrHydA1 C169D	6.6	0.3	60^(Evol)^	NA

a Activity reported as *μ*mol H_2_/min/mg enzyme for either H_2_ oxidation or evolution.

b The pKa of the Serine -O(H) is estimated from values for Serine -O(H) in chymotrypsin analogues (Bruice et al., 1962; Frey, 2001).

c The amount (or fold) change in the variant oxidation/evolution reactivity ratios when compared to the corresponding WT ratio, the direction of the shift (either toward H_2_ oxidation or H_2_ evolution) is indicated in superscripts.

d *E*_m_ is defined as the redox couple between H_ox_ and H_red_ or H_red_H+. CrHydA1 C169S *E*_m_ value at pH 8 (Mulder et al., 2017). CrHydA1 -400 mV (Silakov et al., 2009) and −362 mV (Sommer et al., 2017) determined from separate studies.

### [FeFe]-hydrogenase Reactivity and the pKa, *E*_m_ Scaling Relationship

**Figure 2:**
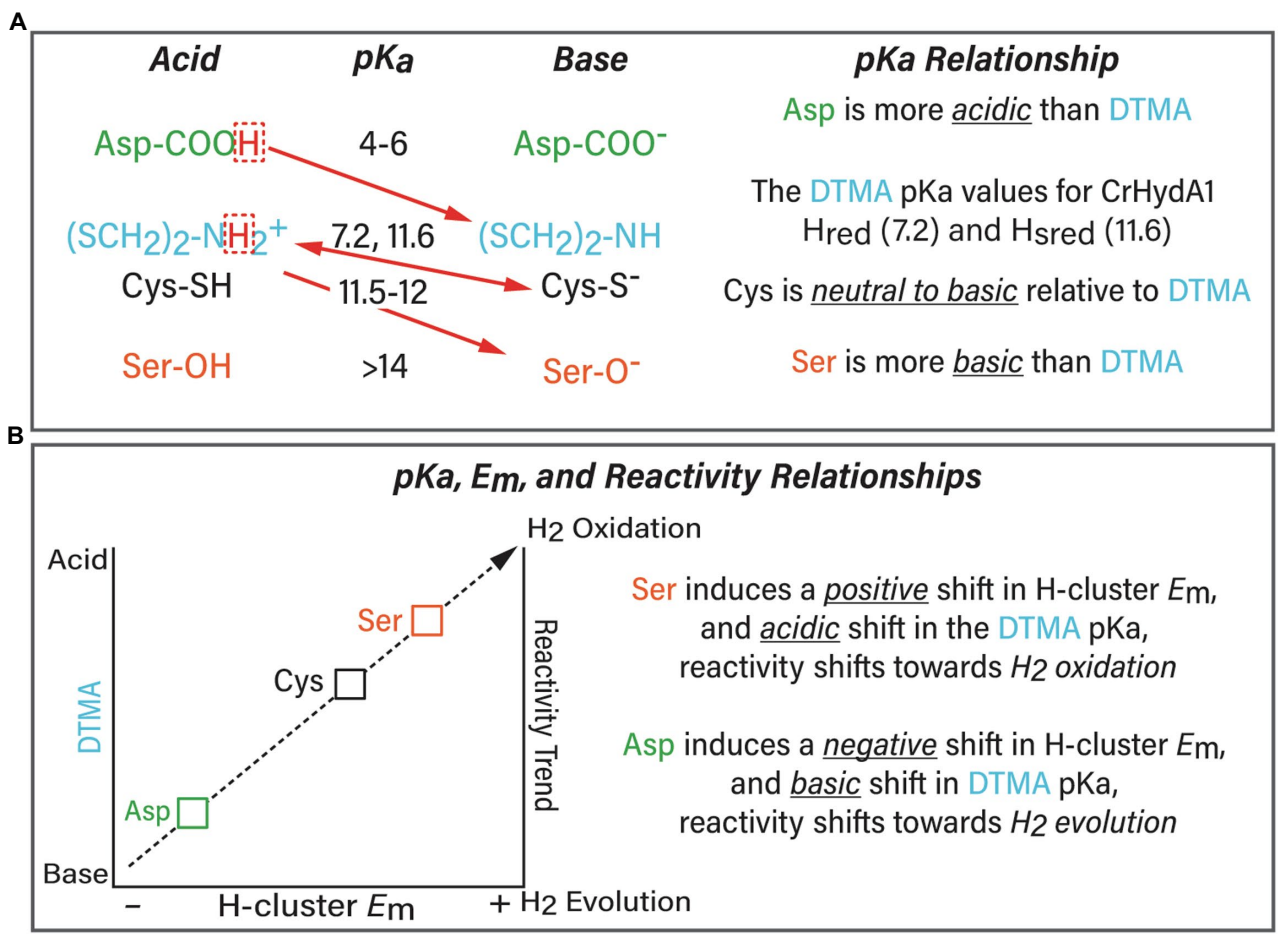
**(A)** Summary of the relationship between the exchange site pKa (Asp, green; Cys, black; and Ser, orange) and pKa of the H-cluster DTMA ligand (blue). The preferred proton transfer direction is indicated with red arrows. The pKa values for Asp, Cys, and Ser were calculated in this study, and the measured pKa values for the bridging ligand di(thiolmethyl)amine (DTMA, blue) were taken from (Sommer et al., 2017; Birrell et al., 2021) for CrHydA1. The scaling model predicts that Asp induces a basic shift whereas Ser induces an acidic shift in DTMA pKa, this would create an even more favorable direction bias than indicated in the Figure. **(B)** Summary of the model of the pKa, *E*_m_, and reactivity relationships based on the results of this work.

The selection of either Cys, Ser, or Asp as the proton exchange site residue imparts a profound control over reactivity, which we propose in part results from perturbing the balance between exchange site pKa (Tables 1–3) and the pKa of the DTMA ligand of the H-cluster (Figure 2), and changes to H-bonding at the DTMA in the variants (Duan et al., 2018; Pham et al., 2018). The change from Cys to Ser also coincides with previous observations (Mulder et al., 2017) of a positive shift in the measured H-cluster reduction potentials, consistent with changes to the H-cluster electronic structure. The values for CrHydA1 C169S compared to WT are −283 and −400 mV vs. NHE for reduction of H_ox_ to H_red_, and −431 and −460 mV vs. NHE for reduction of H_red_ to H_hyd_ or H_sred_, respectively (Silakov et al., 2009; Mulder et al., 2017; Table 4). Conversely, it has been shown that CaI C298D has a proportionately higher population of H_ox_ under reduction with either H_2_ or sodium dithionite compared to WT CaI (Morra et al., 2016). This difference is consistent with a negative shift in the *E*_m_ value, and presumably a shift toward a more basic pKa value of the H-cluster DTMA ligand.

The differential stabilization of H-cluster oxidation states and *E*_m_ shifts can be explained within the framework of the linear-scaling relationships between the pKa and *E*_m_ of a transition metal catalyst (Figure 2; Bullock et al., 2014; Brereton et al., 2020; Puthenkalathil and Ensing, 2021). In this relationship, there is a direct correlation of the metal site pKa to the *E*_m_ value of the reduction potential, where a more acidic pKa is matched by a more positive reduction potential. For the serine variant of CrHydA1, substitution of the more basic serine would be predicted to induce a relative acidic shift in the H-cluster DTMA pKa value, and the *E*_m_ values of the H-cluster would be predicted to shift to more positive values. This is in fact the case (Table 4), which explains in part why the serine variants of CrHydA1 and CaI exhibit higher populations of the reduced states, predominantly H_sred_ and H_hyd_, compared to the native enzyme under the same reducing conditions (Mulder et al., 2014, 2017; Pham et al., 2018; Ratzloff et al., 2018). The corresponding effect is also observed for the CaI aspartate variant, which maintains a high population of H_ox_ under reducing conditions (Morra et al., 2016). Thus, the effect of the cysteine variants on [FeFe]-hydrogenase reactivity can be modeled as a manifestation of combined changes to the pKa relationship with DTMA, and to the *E*_m_ values of the H-cluster, which are summarized in Figure 2. The collective effect leads to a change in reactivity of [FeFe]-hydrogenase for H_2_ oxidation (e.g., acidic H-cluster pKa and positive *E*_m_ shift) or H_2_ evolution (e.g., basic H-cluster pKa and negative *E*_m_ shift; Artz et al., 2020b). The relationship between the exchange site pKa and the H-cluster DTMA pKa modeled in Figure 2 predicts that there should be a corresponding negative shift in the value of *E*_m_ for C→D variants. This prediction is supported by IR spectra of the H_2,_ and dithionite reduced CaI C298D that clearly shows a high population of H_ox,_ and only minor populations of reduced states compared to the spectra of WT CaI, prepared under the same conditions (Morra et al., 2016).

### Expanding the Model for Exchange Site Influence on Reactivity and the Special Case of CpII

It is also possible that the differences in reactivity arise from contributions other than pKa effects. In agreement with this, there are changes in the H-cluster electronic structures of Cys variants evidenced by changes in the FTIR and EPR spectra of catalytic intermediates compared to native enzymes (Mulder et al., 2013, 2014, 2017; Morra et al., 2016), that may occur from secondary sphere effects in the variants. Examples of these effects include the hydrophobicity, charge, electrostatics, and H-bonding networks that can control FeS cluster electronic structures and reduction potentials (Zuris et al., 2010; Hosseinzadeh and Lu, 2016). Indeed, the H_hyd_ state of the CrHydA1 C→S variant has been modeled as having a more contracted H-bonding network between the H-cluster Fe_D_ and the serine O-atom, when compared to the distance from Fe_D_ and the S-atom of cysteine in the native enzyme (Pham et al., 2018). The shorter N–H-O bond lengths vs. N–H-S accounted for shifts in the Fe-H/D frequencies in the NRVS spectra of H_hyd_, and likely account for frequency shifts in *v*CO modes of redox intermediates in the FTIR spectra of the Cys-to-Ser variant versus wild-type (see Mulder et al., 2014, 2017).

Furthermore, whereas the model we set forth in Figure 2 correlates trends in reactivity to changes in pKa, it does not completely account for differences in intrinsic reactivity between the different types of [FeFe]-hydrogenases we tested. Specifically, the fact that the cysteine variants of CpII have comparatively low H_2_ evolution rates indicates that control of reactivity is not entirely embodied within the pKa relationship. The fact that cysteine has a basic pKa in all [FeFe]-hydrogenases, yet CpII is strongly biased toward H_2_ oxidation, implies that the H-cluster may be tuned differently compared to CpI/CaI, or CrHydA1. This could include effects such as dynamic secondary interactions and local electrostatics around the H-cluster, or differential stabilization of catalytic intermediates (Artz et al., 2020b). Mechanisms such as long-range potential effects from F-clusters may also contribute to the differences in reactivity of CpII vs. CpI/CaI and CrHydA1. These details indicate there are additional layers of control of H-cluster reactivity, for example, from the surrounding dielectric (Artz et al., 2020b), spin (de)localization, and other aspects of the electronic structure, the effects of which are being currently investigated by our group.

## Conclusion

The outcome of these results identifies that the exchange site residue in proton transfer at the H-cluster can be used to control the reaction equilibria of [FeFe]-hydrogenases for H_2_ activation. Together, the interplay of pKa and electronic effects integrate as a framework for rationalizing the overall free energy landscape surrounding the H-cluster in terms of a scaling relationship, useful for understanding how [FeFe]-hydrogenase control reactivity (Figure 2). Fine-tuning the H-cluster electronic structure by other outer non-coordinating residues is emerging from biophysical and structural studies of [FeFe]-hydrogenase diversity (Poudel et al., 2016; Caserta et al., 2018; Chongdar et al., 2018; Rodríguez-Maciá et al., 2019; Artz et al., 2020b; Lampret et al., 2020; Fasano et al., 2021) and informed by decades of studies on iron–sulfur clusters (Beinert et al., 1997; Bominaar et al., 1997; Brereton et al., 1998, 1999; Ye et al., 2019). Combining mutational studies with biochemical and biophysical analysis in the context of these findings will likely contribute to further insights into the exquisite control of catalysis and reactivity in [FeFe]-hydrogenases.

## Data Availability Statement

The original contributions presented in the study are included in the article/supplementary material; further inquiries can be directed to the corresponding author.

## Author Contributions

All authors analyzed data respective to their experiments. EK, DM, and PK performed protein expression, purification, and biochemical assays. VB conducted computational analyses on [FeFe]-hydrogenases. All authors contributed to the writing and/or editing the manuscript. All authors contributed to the article and approved the submitted version.

## Funding

Funding was provided by the U.S. Department of Energy Office of Basic Energy Sciences, Division of Chemical Sciences, Geosciences, and Biosciences, Photosynthetic Systems Program.

## Licenses and Permissions

This work was authored in part by the Alliance for Sustainable Energy, LLC, the manager and operator of the National Renewable Energy Laboratory for the U.S. Department of Energy (DOE) under Contract No. DE-AC36-08GO28308.The U.S. Government retains and the publisher, by accepting the article for publication, acknowledge that the U.S. Government retains a nonexclusive, paid-up, irrevocable, worldwide license to publish or reproduce the published form of this work, or allow others to do so, for U.S. Government purposes.

## Conflict of Interest

All authors are employees of Alliance for Sustainable Energy, LLC, an M&O contractor of the U.S. Government.

## Author Disclaimer

The views expressed in the article do not necessarily represent the views of the DOE or the U.S. Government.

## Publisher’s Note

All claims expressed in this article are solely those of the authors and do not necessarily represent those of their affiliated organizations, or those of the publisher, the editors and the reviewers. Any product that may be evaluated in this article, or claim that may be made by its manufacturer, is not guaranteed or endorsed by the publisher.

## Abbreviations

PT: Proton transfer
ET: Electron transfer
H-bonding: hydrogen-bonding
